# Role of the polypeptide N-acetylgalactosaminyltransferase 3 in ovarian cancer progression: possible implications in abnormal mucin O-glycosylation

**DOI:** 10.18632/oncotarget.1652

**Published:** 2014-01-27

**Authors:** Zhi-Qiang Wang, Magdalena Bachvarova, Chantale Morin, Marie Plante, Jean Gregoire, Marie-Claude Renaud, Alexandra Sebastianelli, Dimcho Bachvarov

**Affiliations:** ^1^ Department of Molecular Medicine, Laval University, Québec (Québec), Canada; ^2^ Centre de recherche du CHU de Québec, L'Hôtel-Dieu de Québec, Québec (Québec), Canada; ^3^ Department of Obstetrics and Gynecology, Laval University, Québec (Québec), Canada

**Keywords:** GALNT3, epithelial ovarian cancer, DNA hypomethylation, progression-free survival, microarrays, mucin O-glycosylation

## Abstract

Previously, we have identified the polypeptide N-acetylgalactosaminyltransferase 3 (GALNT3) gene as notably hypomethylated in low-malignant potential (LMP) and high-grade (HG) serous epithelial ovarian tumors, compared to normal ovarian tissues. Here we show that GALNT3 is strongly overexpressed in HG serous EOC tumors as compared to normal ovarian tissue. Moreover, the GALNT3 expression significantly correlated with shorter progression-free survival (PFS) intervals in epithelial ovarian cancer (EOC) patients with advanced disease.

Knockdown of the GALNT3 expression in EOC cells led to sharp decrease of cell proliferation and induced S-phase cell cycle arrest. Additionally, GALNT3 suppression significantly inhibited EOC cell migration and invasion. Gene expression profiling and consecutive network and pathway analyses confirmed these findings, as numerous genes and pathways known previously to be implicated in ovarian tumorigenesis, including EOC tumor invasion and metastasis, were found to be downregulated upon GALNT3 suppression, while some tumor suppressor genes were induced. Moreover, GALNT3 downregulation was associated with reduced MUC1 protein expression in EOC cells, probably related to destabilization of the MUC1 protein due to lack of GALNT3 glycosylation activity. GALNT3 knockdown was also accompanied with increase of the cell adhesion molecules β-catenin and E-cadherin, which are normally suppressed by MUC1 in cancer, thus supporting the role of the GALNT3-MUC1 axis in EOC invasion.

Taken together, our data are indicative for a strong oncogenic potential of the GALNT3 gene in advanced EOC and identify this transferase as a novel EOC biomarker and putative EOC therapeutic target. Our findings also suggest that GALNT3 overexpression might contribute to EOC progression through aberrant mucin O-glycosylation

## INTRODUCTION

Epithelial ovarian cancer (EOC) accounts for 4% of all cancers in women and is the leading cause of death from gynecologic malignancies [1]. Despite treatment improvements, long-term survival rates for patients with advanced disease remain disappointing [2]. The molecular basis of EOC initiation and progression is still poorly understood. To establish novel therapeutic and diagnostic strategies against this deadly disease, it is essential to understand its molecular pathology.

Disruption of normal gene regulation is important for carcinogenesis resulting in loss, or gain of genetic function. Recently, the importance of epigenetic perturbation of gene regulation in cancer [3], including EOC [4], has begun to be more fully appreciated. The most studied epigenetic alteration is DNA methylation, the addition of a methyl moiety to the cytosine-5 position within the context of a CpG dinucleotide, mediated by DNA methyltransferases [3]. DNA methylation patterns are reset early in the embryogenesis and reestablished early during development. After that, they are thought to be relatively stable. In cancer, the physiological regulation of DNA methylation is disrupted leading to drastic changes of the distribution pattern of 5-methylcytosine. The heavy methylation found in the bulk of chromatin is reduced, while the normally unmethylated CpG islands located in the promoter and first exon of genes become hypermethylated. Promoter hypermethylation often leads to inactivation of different tumor-suppressing genes and is associated with many important pathways involved in cancer, such as DNA repair, cell cycle regulation, apoptosis, carcinogen metabolism, hormonal response, and cell adherence [5]. Aberrant DNA methylation is also involved in the development of resistance to chemotherapy (CT) [6]. The role of DNA hypomethylation in carcinogenesis is less studied. Recent studies have demonstrated that global decrease in the level of DNA methylation is related to hypomethylation of repeated sequences, increase in genetic instability, as well as reactivation of proto-oncogenes and pro-metastasis genes (reviewed in [7]).

Similar to other malignancies, aberrant DNA methylation, including global hypomethylation of heterochromatin and local CpG island methylation, occurs in EOC and contributes to ovarian tumorigenesis and mechanisms of chemoresistance [4]. Applying a more global array-based technology, several studies have demonstrated that DNA methylation changes in ovarian cancer are cumulative with disease progression and CT resistance [8–10]. Using a similar approach (methylated DNA immunoprecipitation coupled to CpG island tiling arrays) we have recently shown that DNA hypermethylation occurs in less invasive/early stages of ovarian tumorigenesis, while advanced disease was associated with DNA hypomethylation of a number of oncogenes, implicated in cancer progression, invasion/metastasis and probably chemoresistance [11]. The polypeptide N-acetylgalactosaminyltransferase 3 gene (*GalNAc-T3* gene, also known as *GALNT3*) was among the genes identified to be notably hypomethylated in low-malignant potential (LMP) and high grade (HG) serous EOC tumors [11]. The *GALNT3* gene is a member of the GalNAc-transferases (GALNAC-Ts) gene family; the genes of this family conduct the transfer of N-acetyl galactosamine (GalNAc) to the hydroxyl group of a serine or threonine residue in the first step of *O*-linked oligosaccharide biosynthesis [12]. So far, 20 members of the GALNAC-Ts gene family have been identified and most of them encode an active polypeptide GALNT functioning in the primary step of the *O*-glycosylation of different proteins, including mucins [13]. Aberrant mucin-type *O*-glycosylation represents one of the most abundant posttranslational cancer-associated changes, comprising diverse biologic and pathologic consequences influencing growth and survival of cancer cells and their ability for invasion and metastasis [14]. The membrane-associated mucin-1 *(MUC1)*, as one highly glycosylated protein, is overexpressed in more than 90% of high-grade EOC tumors including metastatic lesions, as *MUC1* expression correlated with EOC progression [15].

This prompted us to further investigate if *GALNT3* displays elevated expression levels in serous EOC tumors with different malignant potential, and whether this gene is functionally implicated in EOC tumorigenesis, including disease progression and response to treatment. Here we present experimental data, which strongly suggest that *GALNT3* is overexpressed HG serous EOC tumors compared to LMP tumors and normal tissues, which probably correlates with its hypomethylated status. We also provide evidence that the *GALNT3* gene is involved in EOC cell proliferation and migration/invasion, due to its possible role in *MUC1* protein stabilization.

## RESULTS

### Overexpression of GALNT3 in HG serous EOC tumors: correlation with progression-free survival

Using an epigenomics approach, we have previously identified the *GALNT3* gene as hypomethylated in LMP and HG EOC tumors, when compared to normal tissues [11]. Here, we further validated the *GALNT3* methylation in independent set of EOC tumors (including LMP and HG tumors) using alternative approach (BSP sequencing). Our BSP analyses confirmed the *GALNT3* hypomethylation status in both LMP (including borderline and Gl) and HG (G3) serous EOC tumors (see Supplemental Figure 1). We consecutively evaluated *GALNT3* protein expression by IHC in serous EOC tumors and ovarian normal tissue samples, using tissue microarrays (TMAs). Our TMAs included triplicate cores of 117 serous EOC tumors, including 13 LMP tumors and 104 HG ovarian tumors. Thirteen normal ovarian tissue samples were also included as controls. Table 1 shows the major clinical characteristics of these patients for whom extensive follow-up clinical data (up to 5-years) were available. The age ranged from 41 to 83 years (median: 66 years). High-grade tumors were all grade 3 (100%) including stage III (69%) and stage IV (31%) tumors. The majority of patients (93%) received a combination of platinum and paclitaxel. The median baseline CA125 was around 800. Forty percent of the patients had a progression or a recurrence within the first 6 months of follow-up; for 39 % of the patients the progression-free survival (PFS) interval was in the range of 7 to 24 months, and 21 % of the patients displayed PFS values higher than 25 months (Table 1).

**Table 1:** Detailed Patients' clinicopathological characteristics

Variable	Range	n/total	%
Age (years)	≥65	64/130	49
	<65	66/130	51
Median age	66		
Tissue/tumor type	Normal	13/130	10
	LMP	13/130	10
	High-grade	104/130	80
Grade	3	104/104	100
Stage	III	72/104	69
	IV	32/104	31
Chemotherapy	platinum+taxol	97/104	93
	Other	13/104	7
CA125	≥800	47/104	45
	<800	53/104	55
PFS (months)*	0–6	41/103	40
	7–24	40/103	39
	>25	22/103	21

* Extended follow-up, including PFS values, were available for 103 patients.

**Figure 1:**
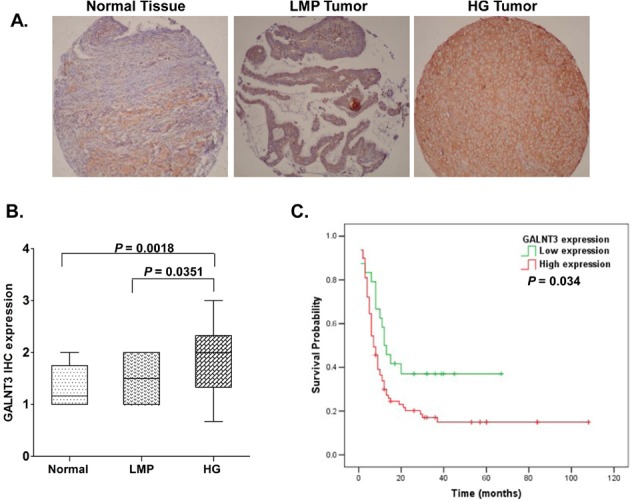
Analysis of *GALNT3* expression in serous EOC tumors by IHC: correlation with PFS A. Representative IHC images of *GALNT3* protein expression in normal ovarian tissues, low-malignant potential (LMP) tumors and high-grade (HG) tumors. B. Box-plot presentation of *GALNT3* protein expression levels in normal ovarian tissues, LMP tumors and HG tumors. C. Kaplan-Meier curves for PFS according to the level of *GALNT3* IHC intensity in tumor samples of 103 serous EOC patients with advanced disease.

As seen from Figure 1, *GALNT3* displayed significantly higher expression only in HG serous EOC tumors, when compared to normal tissues (*p* = 0.0018) and LMP tumors (*p =* 0.035). We further constructed Kaplan-Meier survival curves based on the *GALNT3* expression analyses in the cohort of 103 HG serous EOC patients. Our analyses revealed that *GALNT3* expression displayed significant inverse association with PFS of serous ovarian adenocarcinoma patients with advanced disease; i.e., women with lower *GALNT3* expression had a better survival without progression than those with higher *GALNT3* expression (p=0.034; see Fig. 1C).

### Phenotype analysis of GALNT3 suppression in EOC cells: possible implications in EOC cell proliferation, cell cycle control, migration and invasion

Next, we decided to verify if shRNA-mediated *GALNT3* gene knockdown could produce any cancer-related phenotypic changes in EOC cells. We tested several EOC cell lines for endogenous *GALNT3* protein expression by Western blot analysis (see Figure 8A). Among these, the A2780s cell line displayed strong *GALNT3* expression and was further used to generate stably transfected shRNA-GALNT3 clones. Clone selection for further analyses was based on sqRT-PCR and Western blot validation of the *GALNT3* mRNA/protein expression in selected clones, compared to empty vector-transfected clones. Among the analyzed clones, the shRNA-GALNT3 knockdown clones sh-G1 and sh-G2 displayed a significant decrease of *GALNT3* expression levels compared to the mock-transfected control (Supplemental Figure 2) and were selected for further analyses.

**Figure 2:**
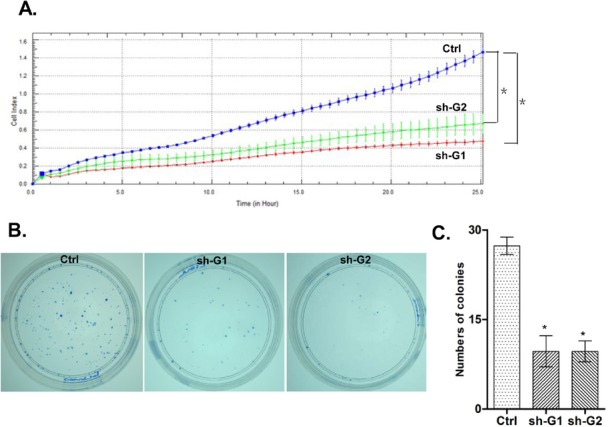
ShRNA-mediated knockdown of the *GALNT3* expression in A2780s cells A. effect of shRNA-GALNT3 knockdown clones 1 and 2 (sh-G1 and sh-G2) on cell proliferation, compared to the control clone (Ctrl); B. Representative images of colony formation assays following *GALNT3* knockdown; C. Quantitative determinations (graph-bars) of data obtained: results are expressed as numbers of colonies formed in the *GALNT3* knockdown clones sh-Gl- and sh-G2 compared to the ctrl clone. Differences were determined using the Student's t-test. Error bars denote mean ± SEM; *indicates statistical significance (*p <* 0.05).

**Figure 3:**
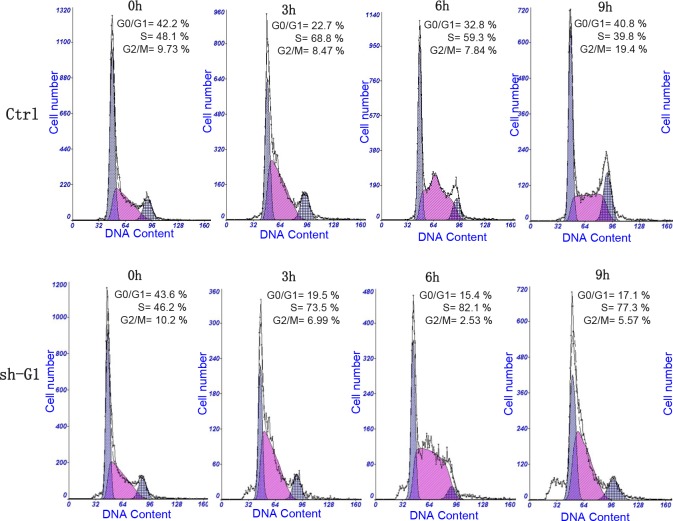
Effect of shRNA-mediated *GALNT3* on cell cycle control in A2780s cells Cell-cycle profile was examined by flow cytometry and percentages of cells in G0/G1, S, and G2/M phase in the shRNA-GALNT3 clone 1 (sh-G1) were compared to the mock-transfected control (ctrl) clone. Propidium iodide staining shows an increased fraction of cells in the S-phase and corresponding decrease of cells in both G1 - and G2/M-phase at 6 and 9 h after removing hydroxyurea in the shRNA-GALNT3 clone Gl (sh-G1), when compared with the control clone (ctrl).

We investigated the impact of the *GALNT3* gene suppression on A2780s cell proliferation, cell cycle control, migration, invasion, and sensitivity to cisplatin and paclitaxel (drugs, conventionally used for first-line EOC CT). The *GALNT3* gene knockdown led to sharp decrease of the number of viable adherent cells (represented by cell index), compared to control cells (Figure 2A). This observation was further supported by the colony formation assay showing that the numbers of clones formed by cells with stably reduced *GALNT3* expression were significantly lower compared to control cells (Figure 2B and 2C). Taken together, our observations strongly indicate an influence of *GALNT3* on EOC cell proliferation and further on their propensity to form colonies. Moreover, when compared with the control clone, the shRNA-GALNT3 clone sh-G1 exhibited a significant accumulation of cells in the S phase at 6 and 9 hours after removing hydroxyurea (Figure 3). These data indicate that *GALNT3* depletion induces S cell cycle arrest which could explain the drastic reduction in the proliferation rates of these cells observed earlier.

**Figure 4:**
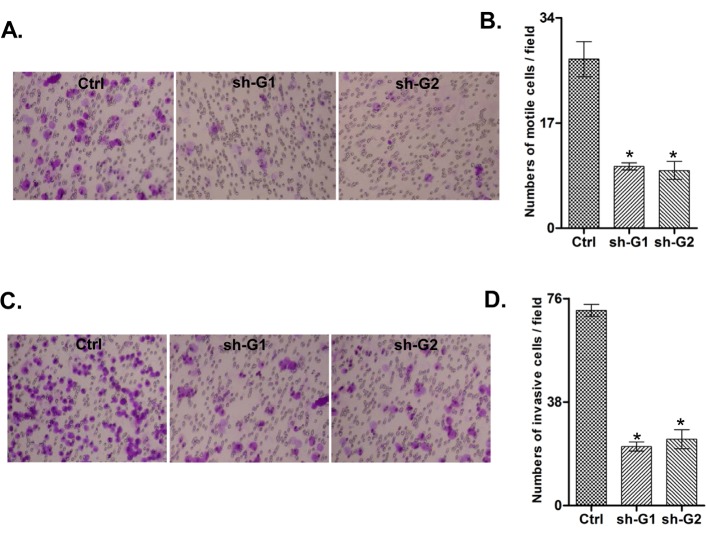
Effect of *GALNT3* knockdown on A2780s cell migration and invasion A. Migration was assessed using Boyden-chamber assay. Cells from the shRNA-GALNT3 clones 1 and 2 (sh-G1 and sh-G2) and the control (ctrl) clone were seeded into the upper chambers in 0.1% FBS containing medium at a density of 2.5 × 10^4^ per well, and 600 μl of 1% FBS containing medium was placed in the lower chamber as a chemoattractant. After 24 h at 37°C in 5% C02, the cells were fixed with cold methanol and stained with blue trypan solution. Migrated cells on the underside of the filter were photographed and counted by phase contrast microscopy. C. Cell invasion was assayed in a similar way, as the upper chambers were coated with Matrigel. Here, NIH3T3 conditioned medium was added in the lower chamber as a chemoattractant (see Materials and Methods for details). All experiments were performed in triplicate. For each experiment, cell number was calculated as the total count from 10 random fields per filter (at magnification ×40). The bar graphs in panels B and D represent quantitative determinations of data obtained by selecting 10 random fields per filter under phase contrast microscopy and results are expressed as % change of the sh-G1 and sh-G2 clones over the ctrl clone. Differences between shRNA-GALNT3-transfected and vehicle-transfected A2780s cells were determined by a Student's *t*-test; error bars denote mean ± SEM; *indicates statistical significance (p<0.05).

Additionally, the *GALNT3* suppression significantly inhibited both migration and invasion of A2780s cells. As shown in Figure 4A and 4B, the numbers of A2780 cells that passed through the filter using the sh-G1 and sh-G2 clones were remarkably less than that in the negative control (ctrl) clone, which is indicative for a role for *GALNT3* in the regulation of invasion and migration in EOC.

Finally, *GALNT3* suppression had no significant impact on A2780s cisplatin and paclitaxel sensitivity (see Supplemental Figure 3).

### Molecular mechanisms of GALNT3 action in EOC cells

To better understand the molecular mechanisms of *GALNT3* action in A2780s cells, we employed the Agilent Whole Human Genome microarrays, containing 44, 000 genes to identify global gene expression changes upon *GALNT3* suppression in A2780s cells. We compared the gene expression of the previously selected shRNA-GALNT3 clones (sh-G1 and sh-G2) against the corresponding control clone (ctrl). All microarray experiments were performed in duplicates, as two hybridizations were carried out for the *GALNT3* suppressing cell clone against the corresponding control, using a fluorescent dye reversal (dye-swap) technique. For each comparison, a subset of differentially expressed genes was selected displaying at least 1.5-fold difference in both duplicate microarray experiments. Using these selection criteria, we found 98 genes were upregulated and 375 were downregulated in A2780s cells upon *GALNT3* knockdown. Table 2 shows a list of selected functionally related groups of genes that were differentially expressed (≥ 1.5-fold) in A2780s cells following *GALNT3* suppression. As seen from Table 2, genes with previously shown implication in mechanisms of cell adhesion, cell development, cell growth … proliferation, transport, immune … inflammatory response, metabolism, regulation of transcription and signal transduction, were predominantly or exclusively suppressed, while *GALNT3* knockdown was related with the induction of some signal transduction-related genes. Supplemental Table 2 shows the complete list of the differentially expressed genes (≥ 1.5-fold) following *GALNT3* knockdown in A2780s cells.

**Table 2:** Selected differentially expressed gene groups in A2780s cells upon GALNT3 knockdown

A. Upregulated genes	
cell adhesion	ITGA1, PCDH17, KITLG, NCAM21, AMIGO2, PCDH20, PCDH10, PCDH7, HAPLN1, NELL2,MASS1, CCR1
metabolism	ARSJ, PLA2G4A, TIPARP, DNAJC12, MEOX1, SMA5, TLL1, BCAT1, HS6ST2 LOXL3, PDZRN3, TRIM7, RNF128
regulation of transcription	ZFHX4, ESRRG, DACH2, TSC22D1, NSBP1
signal transduction	SNX10, CHN1, CENTD1, MGST2, NPY1R SUCNR1, PIK3CA, PPFIBP2, RGS2, AKAP12, RAGE, PRKG1, DNM3, BMP7, DIAPH2, TAOK3, DUSP6, ARRDC3, PRICKLE1, ARRDC3, ARHGAP24
transport	COL3A1, KCNS1, SLC9A9, SLC16A7, SLC40A1, KCNMB4, GPM6A
B. Downregulated genes	
cell adhesion	KERA, THBS1, PALM, KRT8, FLRT3, LAMB1, CXADR, HAPLN3, OMG, PCDH9, CDH2, BAU, plexin C1, NRXN1, NID2, EPDR1, PODXL, COL18A1, COL9A1, COL6A2, SSPN, LUM, TES, KERATIN, FLNC
cell cycle	CCNG2, CDK6, BCL2, HIPK2, APC2, MATK, F2R, IFITM1, MDK, PLCB1
cell development	CALD1, MYH11-SM2, MYH11-SM1, PLXDC2, OLFM1, MEST, ACTG2, ARMCX2, FLJ21159, ARMCX3, troponin T, TNNT2, MYL7, MGC:71510, BTG1, C11orf8, EPB41L3,ARMCX3
cell growth … proliferation	IGFBP2, CTGF, PRSS11, CYR61, IGFBP6, ELF4, DAB2, wdr16, NEFL, COTL1, LOXL1, OLFML3, CK 18, MY05C, INA, SEMA3D, SPOCK2, MYO10, PVRL3, MFAP2, FNDC5, CAV1, CK 18
transport	XK, AP1S2, NPTX1, GRIA3, CP, CHRNA3, SLC30A8, KCNC1, KCNC3, TRPC4, CACNA1G, DKFZp761K0912, KCTD16, SLC43A2, RAB11FIP4, SORT1
immune … inflammatory response	INDO, TAPI, CGREF1, ANKRD1, MMD, GPX7, OAS3, HLA-B, HLA-A, HLA-Cx52, HLA-C, HLA-F, HLA-H, GBP1, PTGER4, IL7, HLA-G, NFKBIZ, IGSF9, CD302, PNMA2, TNMD
metabolism	L-arginine, SORL1, MGC:57495, GLT25D2, TRIM34, TRIM43, TRIM6, TRIM49, HSYRA2001105, TRIM48, SMYD3, SOST, MMP10, MMP3, ADPN, NFIB, RBMS1, ERVK6, NAP1L3, UCHL1, UBE1L, GLDC, SUMF1, MMP1, GLUL, PGDS, FLJ10986, PPP1R1A, GFPT2, CHST6, ME3, ST3GAL1, B3GALT3, PRSS12, PSMB9, LRAP, CA3, AHCY, ACAS2L, GALNT14, FLJ31568, BASP1, FIGN, TRIM51, NUDT10, OSR2, SCG3
regulation of transcription	SOX2, MSX1, COL19A1, POSTN, CART1, ZNF426, ZSCAN4, KIAA0222, FOXA1, TFAP2A, NFE2L3, ISGF3G, ETV7, NR0B1, AR, IFI16, GATA6, GATA5, NR2F2, NFE2, TBX1, MYEF2, LIN28, LHX9, SP100, LHX9, ZNF217, SP140, ARNTL, ZFHX1B, DNAPTP6
signal transduction	DKK3, DPYSL2, GUCY1B3, S100A11, PLCE1, SPP1, BMP2, BMP4, MAP3K15, PFTK1, MYLK, MARK1, EPHB3, PDGFRB, PDGFRA, PRKCB1, PIM1, PTPRE, PLEKHK1, IRS1, CXCR4, GPR84, GCG, BAD, FGF18, FGG, CD53, TAC3, NXPH2, KIAA1493, ARHGEF4, RHOBTB1, RAB3C, BDNF, RGS5, RGS18, SFRP1, MAMDC2, CKB, HRASLS, LOC130576, CKB, GBP4, ANXA3, RAFTLIN, NET02, MGC52057, ASAM, HHIP, ARPP-21, AS AM, mGluR5

**Figure 5:**
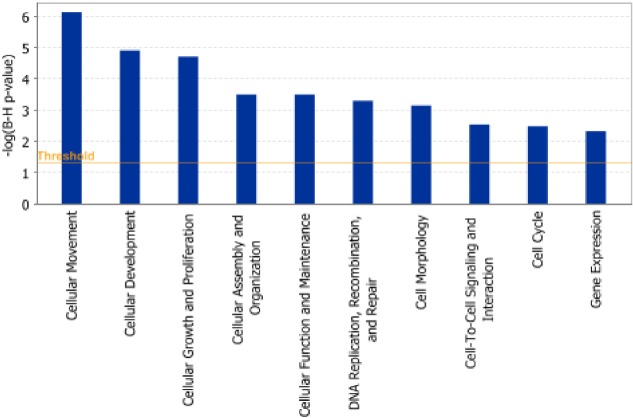
Functional analysis for a dataset of downregulated genes (≥ 1.5-fold) following *GALNT3* suppression in A2780s cells Top functions that meet a Bonferroni-Holm multiple testing correction *p*-value of 0.05 are displayed.

Pathway and network analyses, generated through the use of the IPA software confirmed the major functionally related gene groups, found to be differentially expressed in the *GALNT3* knockdown clones sh-G1 and sh-G2. Upon using a stringent scoring method (the Bonferroni-Holm multiple testing correction *p*-value), we have found a number of pathways to be significantly downregulated following *GALNT3* suppression (including pathways functionally related to cellular movement, cellular development, cellular growth and proliferation, cellular assembly and organization, cellular function and maintenance, DNA replication, recombination, and repair, cell morphology, cell-to-cell signaling and interaction, cell cycle and gene expression; see Figure 5). However, no significant functional pathways were found to be upregulated upon *GALNT3* knockdown when using the same scoring method.

**Figure 6:**
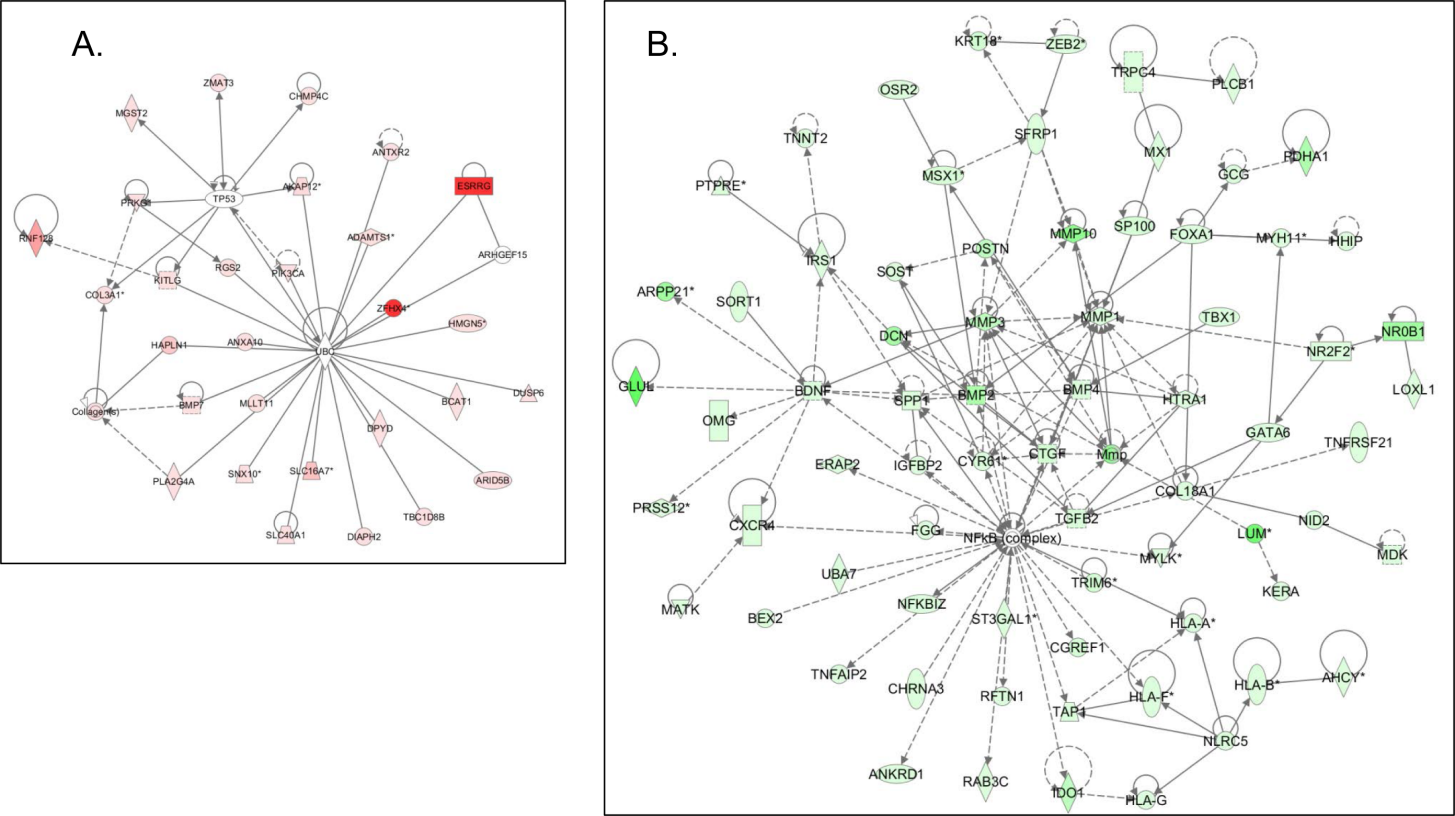
Network analysis of dynamic gene expression in A2780s cells based on the 1.5-fold common gene expression list obtained following shRNA-mediated *GALNT3* knockdown A. Upregulated networks; B. Downregulated networks. The three top-scoring networks (upregulated genes) and the five top-scoring networks (downregulated genes) were merged and are displayed graphically as nodes (genes/gene products) and edges (the biological relationships between the nodes). Intensity of the node color indicates the degree of up- (red) or downregulation (green). Nodes are displayed using various shapes that represent the functional class of the gene product (square, cytokine, vertical oval, transmembrane receptor, rectangle, nuclear receptor, diamond, enzyme, rhomboid, transporter, hexagon, translation factor, horizontal oval, transcription factor, circle, other). Edges are displayed with various labels that describe the nature of relationship between the nodes:__binding only, → acts on. The length of an edge reflects the evidence supporting that node-to-node relationship, in that edges supported by article from literature are shorter. Dotted edges represent indirect interaction.

Common networks obtained upon merging the top-scoring networks recognized some important gene nodes and genes that are specifically up- or downregulated upon *GALNT3* suppression in A2780s cells (Figure 6). Thus, genes and associated interaction partners that were upregulated upon *GALNT3* knockdown (displayed on Figure 6A) comprised members of the ubiquitin C *(UBC)* and tumor protein p53 *(TP53)* interaction networks, including genes, predominantly implicated in signal transduction *(ANTXR2, ANXA10, ARHGEF15, BMP7, DUSP6, KITLG, PIK3CA, PRKG1, RGS2)* and metabolism *(ARID5B, BCAT1, DPYD, ESRRG, HMGN5, MGST2, PLA2G4A, RNF128, ADAMTS1, UBC)*. Major gene nodes that were downregulated upon *GALNT3* knockdown in A2780s cells are presented in Figure 6B; these were mostly involved in metabolism (*MMP1, MMP3, MMP10, SPP1, HTRA1, FOXA1*), signal transduction *(BMP2, BMP4, CYR61, TGFB2, BDNF, POSTN, IRS1, IGFBP2)* and cell growth … proliferation *(CTGF, COL18A1, SFRP1)*.

### Validation of microarray findings with quantitative PCR (qPCR)

**Figure 7:**
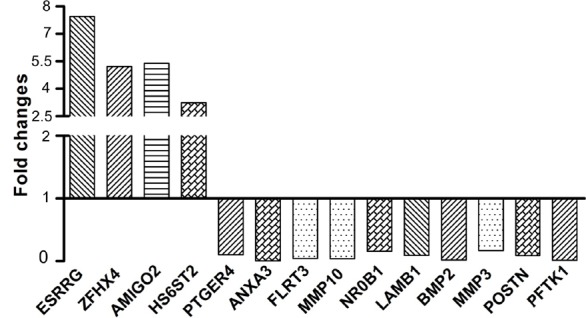
Quantitative PCR validation of microarray results The figure shows bar graphs presentation of the differential expression of the selected genes in A2780s cells following shRNA-mediated *GALNT3* suppression compared to control (vehicle transfected) A2780s cells. The relative copy number was calculated based on the target *gene/18S* ribosomal RNA ratio. Values more than or equal to 1 represent gene upregulation and more than 1 display gene downregulation. The analysis confirmed higher levels of expression for *ESSRRG, ZFHX4, AMIG02* and *HS6ST2* and lower levels of expression for *PTGER4, ANXA3, FLRT3, MMP10, NROB1, LAMB1, BMP2, MMP3, POSTN* and *PFTK1* upon *GALNT3* knockdown.

To validate microarray results, we arbitrarily selected 14 differentially expressed genes and quantified their expression by qPCR in A2780s cells following shRNA-GALNT3 knockdown compared to control (vehicle transfected) A2780s cells. Figure 7 summarizes the gene expression measurements of all validated genes. We found that both methods (microarray analysis and qPCR) detected similar patterns for the up- and down-regulated genes selected for validation.

### Effect of GALNT3 knockdown on MUC1 O-glycosylation and stabilization

**Figure 8:**
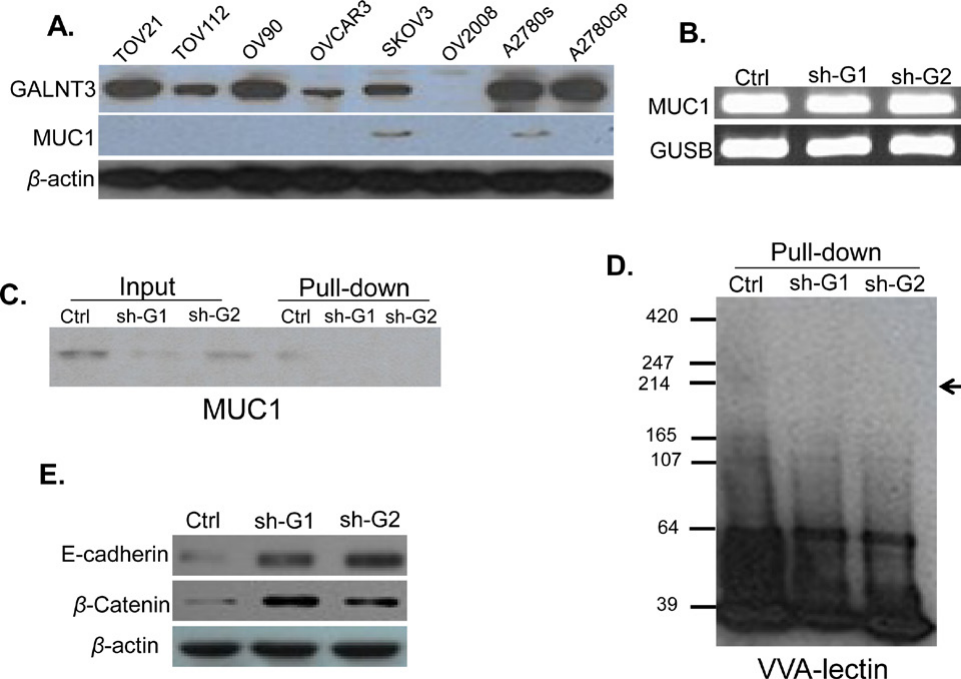
Analysis of GALNT3-mediated *MUC1* glycosylation in EOC cells A. Western blot analysis of *GALNT3* and *MUC1* endogenous protein expression in different EOC cell lines, including A2780s; β-actin was used as a loading control; B. Semi-quantitative RT-PCR analysis of MUC1 mRNA levels in the control clone (ctrl) and shRNA-*GALNT3* clones 1 and 2 (sh-G1 and sh-G2); the *GUSB* gene was used as an internal control; C. Western blot analysis of *MUC1* expression in the ctrl, the sh-G1 and the sh-G2 A2780s clones before (input) and following pull-down assay using biotin-conjugated VVA lectin and streptavidin agarose (pull-down); D. VVA-lectin-mediated immunoblot analysis of GalNAc-conjugated proteins in protein lysates of the ctrl, the sh-G1 and the sh-G2 A2780s clones following VVA lectin pull-down assay (pull-down). The arrow indicates bands corresponding to possible GalNAc-conjugated *MUC1* peptides; E. Western blot analysis for E-cadherin and β-catenin expression in the ctrl, the sh-G1 and the sh-G2 A2780s clones; β-actin was used as a loading control

To further elucidate the *GALNT3* potential oncogenic potential in EOC, we examined the effect of *GALNT3* suppression on the *MUC1* mRNA and protein expression in A2780s cells. We initially analyzed the endogenous *MUC1* protein expression levels in different EOC cells and found that *GALNT3* and *MUC1* proteins were co-expressed in the cell lines A2780s and SKOV3 (Figure 8A). Using a semi-quantitative RT-PCR, we found no differences in the *MUC1* mRNA expression levels between the shRNA-GALNT3 knockdown A2780s clones (sh-G1 and sh-G2) and the corresponding control (Ctrl) A2780s clone (Figure 8B). However, *MUC1* displayed considerably lower protein expression levels in the sh-G1 and sh-G2 clones, compared to the ctrl clone (Figure 8C/input). Moreover, VVA lectin pull-down assay for glycosylated proteins was able to detect a MUC1 protein-specific 200 kDa band only in the ctrl clone, but not in the sh-G1 and sh-G2 clones (Figure 8C/pull-down). Similarly, VVA lectin blot following VVA lectin pull-down assay detected GalNAc-conjugated proteins in the range of 165 — 247 kDa exclusively in the ctrl clone (Figure 8D). Taken together, our findings imply that *GALNT3* gene may contribute to ovarian carcinogenesis through *O*-glycosylation and stabilization of the *MUC1* oncoprotein and possibly, other mucins. Moreover, because downregulation of MUC1 in cancer cells can inhibit cell migration by promoting the expression of the cell adhesion molecules E-cadherin and β-catenin [16], we also investigated the impact of *GALNT3* knockdown on E-cadherin and β-catenin protein expression. As seen from Figure 8E, *GALNT3* suppression remarkably augmented the proteins of both these cell adhesion molecules, suggesting that the involvement of the *GALNT3-MUC1* pathway in EOC invasion could include the destabilization of the E-cadherin/β-catenin complex formation.

## DISCUSSION

The mechanisms for the tumorigenesis, progression and biological aggressiveness of EOCs have not been yet fully clarified. We have recently identified *GALNT3* gene as a novel potentially hypomethylated target in epithelial ovarian cancer (EOC) by our MeDIP-array experiments and consecutively determined that a 315 nt *GALNT3* putative gene control region (located 497 nt downstream of the transcription initiation site, and adjacent to some important control motifs of the *GALNT3* promoter domain [17]) was significantly hypomethylated in LMP and HG serous EOC tumors, compared to normal tissues [11]. In the present study we demonstrate that *GALNT3* is overexpressed exclusively in HG serous ovarian tumors as compared to LMP tumors and normal ovarian tissues; furthermore, we found that *GALNT3* expression was significantly associated with PFS in the studied cohort of 103 HG serous EOC patients *(p =* 0.034; see Fig. 1C). Additionally, our functional analyses are strongly indicative for evident oncogenic capacity of *GALNT3* in serous EOC, including its potential role in EOC cell proliferation, cell cycle control and cell migration/invasion (see Figs. 2–4). However, our findings suggest that epigenetic mechanisms associated with aberrant DNA hypomethylation might not play important role in controlling the *GALNT3* expression in EOC, since we found no correlation between *GALNT3* hypomethylation status and its expression in LMP tumors.

To better elucidate the molecular mechanisms and biological pathways implicated in GALNT3-mediated action in EOC cells, we used a complementary gene expression profiling using the DNA microarray technology to monitor cellular changes in gene expression and discover the molecular targets upon *GALNT3* suppression. To our knowledge, the present work represents the first effort to define global changes in gene expression upon modulation of *GALNT3* gene expression in epithelial cancer cells. The gene expression data and consecutive IPA network and pathway analyses were quite confirmatory of the data obtained by the *GALNT3* functional assays. Indeed, microarray data sustained *GALNT3* correlation with EOC cell proliferation (including cell cycle control), migration and invasion, since *GALNT3* knockdown resulted in reduced expression of genes associated with cell proliferation, cell movement/invasion, and cell cycle control (see Table 2 and Figure 5).

IPA network analysis was indicative for some important gene nodes linked to *GALNT3* suppression in EOC cells, as most of these substantiate and/or complement the functional data obtained. Thus, *GALNT3* knockdown resulted in upregulation of different members of the *p53* and *UBC* interaction networks, both associated with tumor suppressor activities (Figure 6A). Indeed, *p53* is one of the most studied and well characterized tumor suppressor gene in the cancer research field [18], while the *UBC* interaction network and its members were shown to decrease in anchorage-independent cell growth and increase apoptosis, suggesting *UBC* may act as a negative regulator of skin carcinogenesis [19]. Interestingly, the p53/UBC interacting partner *AKAP12* has been characterized as a tumor suppressor gene in many tumor types, acting as an inhibitor of the PKC-Raf/MEK/ERK pro-metastatic pathways [20].

In parallel, upon *GALNT3* knockdown, we have observed predominant and strong downregulation of major gene nodes known to be implicated in EOC tumorigenesis (*SPP1, CYR61, IRS1, IGFBP2*) [21–24], including EOC dissemination/metastasis *(MMP1, MMP3, MMP10, BMP2, BMP4, TGFB2, BDNF, POSTN)* [25–33]; (see Figure 6B). Additionally, *GALNT3* suppression was associated with downregulation of numerous genes from the *NFkB* pathway (Figure 6B), representing one of the major oncogenic pathways implicated in EOC progression [34] Thus, our findings are strongly indicative for the oncogenic functionality of *GALNT3* in EOC carcinogenesis. These results were not unexpected since recent studies suggested that *GALNT3* overexpression is predominantly associated with more aggressive tumor behavior and poor outcome in various types of cancer [17, 35–39]. However, *GALNT3* expression is also shown to be a negative indicator for disease prognosis in non-small cell lung cancer and colorectal carcinoma [40, 41], including colon carcinoma cells selected for hepatic metastasis [42]. Moreover, dysfunctional *GALNT3* gene causes the rare autosomal recessive metabolic disorder familial tumoral calcinosis [43]. The above data suggest that *GALNT3* can display distinct oncological roles in different cancer types.

The various genes encoding GALNAC-Ts are differentially expressed in malignant tissue compared to normal tissue and were consistently shown to play distinct roles in cancerogenesis and tumor metastasis. Thus, *GALNT6* and *GALNT13* display elevated levels in different cancer types, including breast and gastric carcinomas *(GALNT6)* and lung cancer and neuroblastoma *(GALNT13)*, as both genes are found to be associated with tumor invasion and metastasis [44–47]. Similarly, *GALNT14* knockdown significantly suppresses the cell migration and altered cellular morphology of EOC cells [48], and *GALNT1* knockdown inhibits proliferation and tumor growth of bladder cancer cells [49], while *GALNT10* expression positively correlates with histological type and degree of differentiation of gastric cancer [50]. In contrast, *GALNT2* exerts anti-proliferative and anti-metastatic activity in gastric, brain and hepatocellular carcinomas [51–53], while *GALNT9* is positively associated with a better clinical outcome in neuroblastoma patients [54], and *GALNT12* expression represents a negative marker especially of metastatic gastric and colorectal cancer [55]. Also, *GALNT14* expression correlates with pro-apoptotic drug's sensitivity in pancreatic carcinoma, non-small-cell lung carcinoma and melanoma cell lines [56]. Interestingly, elevated *GALNT7* expression contributes to growth and invasiveness of cervical cancer cells [57], while miR-30b/30d targeted *GALNT7* suppression promotes the metastatic behavior of melanoma cells [58], which is indicative for the contrasting mode of action of this transferase in different tumor types. Similarly, *GALNT6* expression in pancreatic cancer is associated with better overall survival [59]. The above data suggest that the different isoforms of the human GALNAC-Ts family could have distinct implication in tumorigenesis that could be specific for each cancer type.

The altered expression of different members of the GALNAC-Ts gene family in different malignancies makes them potential biomarkers [60]. Because aberrant glycoproteins as a result of these enzymes may be involved in promoting tumor invasion and metastasis, these enzymes could also be used as therapeutic targets. Indeed. GALNAC-Ts are responsible for the *O*-glycosylation of mucins, as this post-translational modification is critical for their function to protect and control the local environment of the cell surface [61], whereby aberrant glycosylation of mucins can contribute to cell differentiation, adhesion, invasion, and metastasis [62]. *MUC1*, as one highly glycosylated protein, is aberrantly overexpressed in the majority of epithelial cancers [63], including EOC [15]. *MUC1* overexpression in EOC contributes to EOC cell-cell adhesion, proliferation, migration and metastasis and *MUC1* has been identified as a promising target for EOC therapy [64]. It has been demonstrated that *GALNT3* expression is associated with the differentiation or biological behavior of human adenocarcinomas through the initial glycosylation of mucin-type *O*-linked glycoproteins [65]. Since *MUC1* glycosylation could also be regulated by *GALNT3* [66], we decided to verify if *GALNT3* suppression could affect *MUC1* expression in EOC cells. We were able to show that knockdown of *GALNT3* protein induced the reduction of *MUC1* protein in EOC cells although the transcriptional level of *MUC1* was unchanged (Figure 8B and C). Moreover, by using VVA lectin pull-down assay and consecutive VVA lectin blotting analysis, we found significantly diminished protein expression of glycosylated proteins in EOC cells upon *GALNT3* suppression (Figure 8D), suggesting that *GALNT3* may influence the post-translational modification and stabilization of *MUC1* and other glycosylated proteins in EOC cells. Our data are also indicative for the possible implication of the *GALNT3-MUC1* axis in EOC cell invasion via the regulation of expression of the cell adhesion proteins E-cadherin and β-catenin. Interestingly, *GALNT6* has shown rather similar effects on *MUC1* expression/glycosylation in breast cancer cells [44]; this is not quite unexpected, given that human *GALNT3* and *GALNT6* transferases display very high similarity in DNA and amino acid sequence throughout the coding region, as well as and similar kinetic properties that are distinct from all other human GALNAC-Ts [67].

In conclusion, we have shown that the *GALNT3* transferase is significantly overexpressed in HG serous EOC tumors compared to LMP tumors and normal ovarian tissues, as the *GALNT3* expression correlated with poor prognosis of EOC patients with advanced disease. Consecutive functional analyses of *GALNT3* in EOC cells pointed towards its association with EOC cell proliferation (including cell cycle control), migration and invasion. Gene expression profiling and associated network and pathway analyses confirmed these findings, as numerous genes and pathways known previously to be implicated in ovarian tumorigenesis, including EOC tumor invasion and metastasis, were found to be suppressed upon *GALNT3* knockdown, while some tumor suppressor genes were found to be induced. Moreover, *GALNT3* downregulation was associated with reduced *MUC1* protein expression in EOC cells, related probably with destabilization of the *MUC1* protein due to lack of *GALNT3* glycosylation activity, which results in consecutive accumulation of cell adhesion molecules such as E-cadherin and β-catenin. Hence, *GALNT3* suppression could inhibit cell proliferation and invasion/migration by destabilizing *MUC1* and possibly other mucin-type (*O*-linked) glycoproteins and thus inducing the expression of cell adhesion proteins, although further in-depth analysis will be required to elucidate the precise mechanism of the GALNT3-MUC1 pathway in EOC cells. Our data specify some of the putative mechanisms of abnormal glycosylation in ovarian carcinogenesis and the identification of the *GALNT3* enzyme as novel EOC biomarker and/or possible molecular target for EOC therapy. Further studies are needed to more completely elucidate the functional implications of *GALNT3* and possibly, of other members of the GALNAC-Ts gene family in ovarian tumorigenesis.

## METHODS

### Patients and tissue specimens

Snap frozen and formalin-fixed paraffin-embedded (FFPE) tissues of 117 EOC tumors were obtained at the Hotel-Dieu de Quebec Hospital, Quebec, Canada. These included 13 borderline, or LMP tumors and 104 HG adenocarcinomas. None of the patients received CT before surgery (see Table 1 for detailed clinicopathological characteristics). All tumors were histologically classified according to the criteria defined by the World Health Organization [68]. The CT treatment was completed for all patients and the response to treatment was known. Disease progression was evaluated following the guidelines of the Gynecology Cancer Intergroup [68]. Progression free survival (PFS) was defined as the time from surgery to the first observation of disease progression, recurrence or death. Thirteen normal ovarian samples were derived from women subjected to hysterectomy with oophorectomy due to non-ovarian pathologies. The study was approved by the Clinical Research Ethics Committee of the Hotel-Dieu de Quebec Hospital and all patients signed an informed consent for voluntary participation.

### Cell cultures

The EOC cell lines OVCAR3 and SKOV3 were purchased from American Tissue Type Collection (Manassas, VA); OV-90, OV2008, TOV-112 and TOV-21 cell lines were a kind gift from Dr. Anne-Marie Mes-Masson (Montreal University), while A2780s and A2780cp cell lines were a kind gift from Dr. Benjamin Tsang (Ottawa University). The cell lines were passed in different culture media supplemented with 10% fetal bovine serum, as described previously [69].

### Bisulfite sequencing PCR (BSP) analysis

BSP analysis was performed, as previously described [11]. Briefly, genomic DNAs from normal ovarian tissues, borderline (BL, also known LMP), grade 1 (G1) and grade 3 (G3) EOC tumor specimens were isolated using the Qiagen DNeasy Blood and Tissue Kit. Bisulfite modification of genomic DNAs was done using the Methyl Detector kit (Active Motif, Carlsbad, CA). For BSP, a 315-bp fragment was amplified using primer pairs specific for bisulfite-modified sequences but not harboring CpGs, located at nt + 304 (GATTTTGAGAGGGAGGGT) to nt + 619 (ATCAAAAAAATAAACCCAACTCT) downstream of the GALNT3 transcription start (ATG) codon. BSP primer selection was performed using the Methyl Primer Express Software vl.0 (Applied Biosystems). PCR was done for 30 cycles (94°C, 45 s; 60°C, 45 s; 72°C, 45 s). PCR products were sent for dideoxy-sequencing analysis at the Genomics Analysis Platform at Laval University (http://www.bioinfo.ulaval.ca/seq/en/).

### Tissue microarrays (TMAs) construction and immunohistochemistry (IHC)

TMAs were constructed, as previously described [69–71]. Briefly, one representative block of each ovarian tumor and normal ovarian tissue was selected for the preparation of the tissue arrays. Three 0.6 mm cores of tumor were taken from each tumor block and placed, 0.4 mm apart, on a recipient paraffin block using a commercial tissue arrayer (Beecher Instruments, Sun Prairie, WI). The cores were randomly placed on one of two recipient blocks to avoid IHC evaluation biases. Four micron thick sections were cut for the hematoxylin-eosin (HE) staining and IHC analyses.

IHC was performed, as previously described [69–71]. Briefly, 4 μm tissue sections were deparaffinized and then heated in an autoclave for 12 min to retrieve the antigenicity before blocking with endogenous peroxidase. Following treatment with 3% H_2_0_2_ for 10 min to quench the endogenous peroxidise activity, sections were incubated with the *GALNT3* mouse monoclonal antibody (Abgent, Inc.) at dilution 1:50. Sections were then incubated with a biotinylated secondary antibody (Dako, Carpinteria, CA) and then exposed to a streptavidin complex (Dako, Carpinteria, CA). Complete reaction was revealed by 3-3' diaminobenzidine and slides were counterstained with hematoxylin. *GALNT3* protein expression was assessed by semiquantitative scoring of the intensity of staining and recorded as absent (0), weak (1+), moderate (2+) or strong (3+). The relationship between *GALNT3* expression in serous ovarian carcinomas and normal ovarian tissues was evaluated by the Wilcoxon two-sample test. A significant association was considered when *p*-value was below 0.05. A Kaplan-Meier curve and the log-rank test were performed based on PFS values to test the effect of the intensity of *GALNT3* (3, 2 versus 0, 1) on disease progression.

### Short Hairpin RNA (shRNA) — mediated GALNT3 knockdown in A2780s cells

A shRNA, targeting the GALNT3 sequence 5'-TACTGCTGAAGGAAATCAT-3', was designed using the siRNA Ambion Target Finder software (http://www.ambion.com/techlib/misc/siRNA_finder.html), and subcloned into the pSilencer 4.1 puro vector (Ambion). A2780s cells were stably transfected with the shRNA-GALNT3 plasmid using the ExGen 500 transfection reagent (Fermentas Canada Inc., Burlington ON), according to the manufacturer's instructions. Cells were consecutively grown for 2 weeks in selection medium containing 5 μg/ml puromycin (Wisent, Canada) to isolate stable clones. Cells were also mock-transfected with the pSilencer 4.1 puro vector, and the stably-transfected clones were isolated as controls. Stable clones with inhibited GALNT3 expression were evaluated and validated by semi-quantitative RT-PCR and Western blot.

### Cell proliferation assays using impedance measurement with the xCELLigence system

Cell proliferation (cell index) was checked by the xCELLigence Real-Time Cell Analyzer (RTCA) instrument, as previously described [69, 71]. Cells were seeded in triplicate at 2 × 10^4^ cells/well in the E-Plate 16, a specialized 16-well plate used with the RTCA instrument. Each of the 16 wells on the E-Plate 16 contains an integral sensor electrode array so that cells inside each well can be monitored and assayed. Cell growth was monitored for 24 hours.

### Colony formation assay

Colony formation assay was performed as previously described [69, 71]. A2780s cells were seeded at 500 cells per 60 mm culture dish. After 14 days, the dishes were washed twice in PBS, fixed with cold methanol, stained with Coomassie Blue (Sigma-Aldrich) for 5 min, washed with water and air dried. The number of colonies was determined by imaging with a Multimage™ Cabinet (Alpha Innotech Corporation) and using AlphaEase Fc software.

### Cell migration and invasion assays

Cell migration assays were performed in a modified Boyden-chamber assay using a Transwell chamber insert (6.5 mm diameter) separated by a polycarbonate filter of 5 μm pores (Costar, Cambridge, MA), as previously described [69, 71]. Briefly, shRNA-GALNT3 clones (sh-G1 and sh-G2) and the control (ctrl) were seeded into the upper chambers in 0.1% FBS containing medium at a density of 1.5 × 10^4^ per well, and 600 μl of 1% FBS containing medium was placed in the lower chamber as a chemoattractant. After 5 h at 37°C in 5% CO_2_, the cells were fixed with cold methanol (15 min) and stained with trypan blue solution (5 mins). Cells on the upper surface of the filter were removed with cotton buds. Migrated cells on the underside of the filter were photographed and counted by phase contrast microscopy, by selecting 10 random fields per filter (at magnification × 40). The experiments were performed in triplicate. Cell invasion was assayed in a similar way, as the 8-μn pore polycarbonate filters were coated with 40 μl of Matrigel™ at concentration of 0.5 mg/ml (BD Biosciences, Franklin Lakes, NJ). Here, 600 μl of NIH3T3 conditioned medium was added in the lower chamber as a chemoattractant. Differences between shRNA-GALNT3 clones (sh-G1 and sh-G2) and the control (ctrl) were determined by a Student's *t*-test, where *p <* 0.05 was considered significant.

### Flow cytometry

Flow cytometry analysis was performed, as previously described [69]. Briefly, 7.5 × 10^4^ A2780s cells were treated with 20 mM hydroxyurea (Sigma) for synchronization at the G1/S boundary. After 16 h of incubation, cells were washed once with PBS, and resuspended in 1 ml of complete media. Then, cells were harvested by trypsinization at 0, 3, 6, and 9 h, washed three times with PBS, and fixed with ice-cold 95% ethanol overnight. Cells were washed with PBS (3x) and incubated with propidium iodide (50 μg/ml) (Sigma) in the dark at room temperature for 30 min. Flow cytometric analysis was performed on a Beckman Coulter EPICS XL-MCL analyzer. The cell cycle phase distribution was calculated from the resultant DNA using the cell QuesPro software.

### MTT (cytotoxicity) assay

The MTT cell proliferation assay (Sigma) was used to measure the cell growth inhibition effects of cisplatin and paclitaxel in A2780s cell clones suppressing *GALNT3*, as previously described [69]. Briefly, cell suspensions (at 2 × 10^4^ cells/ml) were transferred to 96-well plates in triplicates and incubated for 3 days with different cisplatin and paclitaxel concentrations (ranging between 1 nM and 100 μM). Then, 20 μl of 3-[4, 5-dimethylthiazol-2-yl]-2, 5-diphenyl-tetrazolium bromide (MTT, 5 mg/ml) was added to each well 4 h before the end of the incubation. After centrifugation and removing the supernatant, 200 μl of dimethyl sulphoxide (DMSO) were added to resolve the crystals and the optical density was measured by microplate reader at 595 nm.

### Gene expression profiling and data analysis

Gene expression analysis was carried out as previously described [69–71]. Briefly, total RNA was extracted from the shRNA-GALNT3 knockdown clones sh-G1 and sh-G2, and the corresponding control (mock-transfected) A2780s clone (ctrl). The quality of the RNA samples was examined by capillary electrophoresis using the Agilent 2100 Bioanalyzer (Agilent). Fluorescently labeled cRNA targets were generated from 0.5 μg of total RNA from each corresponding A2780s cell clone, using the Fluorescent Linear Amplification Kit (Agilent) and 10 mM Cyanine 3- or 5-labeled CTP (PerkinElmer), and following user's manual. Cyanine labeled cRNA from the clone suppressing GALNT3 (sh-G1 and sh-G2) was mixed with the same amount of reverse-color cyanine-labeled cRNA from the corresponding control (ctrl) clone and hybridized on the Agilent Whole Human Genome microarrays, containing 44,000 genes. Array hybridization, washing, scanning, data extraction and analyses were performed as previously described [69]. Network analysis of the microarray data was completed using the Ingenuity Pathway Analysis (IPA) software (see http://www.Ingenuity.com). The microarray data have been deposited to the GEO database (http://www.ncbi.nlm.nih.gov/geo/) with accession number GSE52602.

### Semi-quantitative RT-PCR (sqRT-PCR)

Analysis of GALNT3 gene expression in stably transfected GALNT3 knockdown clone (sh-G1) and the corresponding mock-transfected A2780s clone (ctrl) was performed by sqRT-PCR as previously described [69–71]. The GUSB gene was used as an internal standard. Comparative signal intensity was evaluated using the ImageJ software (http://rsb.info.nih.gov/ij/). Primers were designed for these loci with the sequences freely available from the Entrez Nucleotide database and the Primer3 algorithm for primer design (http://www-genome.wi.mit.edu/cgi-bin/primer/primer3_www.cgi) (see Supplemental Table 1 for sqRT-PCR primer description).

### Quantitative PCR (qPCR)

Quantitative PCR was performed as previously described [71]. Briefly, total RNA was extracted by RNeasy Plus Mini Kit (QIAGEN) and cDNA was obtained by qScript™ cDNA SuperMix (Quanta BioSciences, Inc.). Primers were designed for these loci with the sequences freely available from the Entrez Nucleotide database and the Primer3 algorithm for primer design (http://www-genome.wi.mit.edu/cgi-bin/primer/primer3_www.cgi). The primers used for qPCR validation are listed in Supplemental Table 1. PerfeCTa® SYBR® Green FastMix® (Quanta BioSciences, Inc.) was used according to manufacturer's instructions. PCR reactions were performed on Rotor-Gene RG-3000 Real Time PCR System (Qiagen), with 18S ribosomal RNA used as endogenous control. PCR volume was 20 μl (36-well plate), and conditions were as follow: initial cycle 50°C, 2 min, 95°C, 15 min; 45 cycles at 95°C, 20 s, 60°C, 20 s and 72°C, 20 s; final cycle 72°C, 30 s. Data were analyzed by the Rotor-Gene software using the comparative ΔΔCt method. The relative copy number was calculated based on the target gene/18S RNA ratio.

### Western blotting

Western blot analysis was performed as previously described [69–71]. Briefly, protein lysates were prepared by resuspending cell pellets in Laemmli sample buffer containing 5% *β*-mercaptoethanol. Protein lysates were separated by 6 to 12% Tris-glycine gel electrophoresis and transferred onto a polyvinylidene difluoride membrane using a semidry apparatus (Bio-Rad Laboratories, Hercules, CA). The membrane was blocked with 5% nonfat dry milk in TBST (20 mmol/L Tris-HCl, 0.5 M NaCl, and 0.1% Tween 20), incubated with the anti-GALNT3 mouse monoclonal antibody (1:500) (Abgent, Inc.), anti-MUCl mouse monoclonal antibody (1:500) (Santa Cruz Biotechnology), 0.5 μg/mL of biotin-conjugated VVA lectin (EY Laboratories), anti-E-cadherin rabbit polyclonal antibody (1:500) (Santa Cruz Biotechnology), anti-β-catenin mouse monoclonal antibody (1:500) (Santa Cruz Biotechnology), or anti-β-actin antibody (1:5000) (Santa Cruz Biotechnology), at 4°C overnight. After 3 × 15 min washes with TBST (20 mmol/L Tris-HCl, 0.5 M NaCl, and 0.1% Tween 20) at room temperature, the membrane was incubated with horseradish peroxidase-conjugated goat anti-mouse IgG (Santa Cruz Biotechnology) or 100 ng/ml streptavidin-horseradish peroxidase (Invitrogen) in TBST containing 5% non-fat dry milk for 1–2 h at room temperature. Upon washing, the signal was visualized using ECL solution (Thermo Fisher Scientific, Waltham, MA) and detected on blue sensitive autoradiography film (Marsh Bio Products, Rochester, NY).

### VVA lectin pull-down assay for O-glycosylated (GalNAc-conjugated) proteins

Lectin extracted from Vicia villosa (WA lectin) displays high affinity for GalNAc [72] and has been previously used for pull-down assays to detect GalNAc-conjugated proteins [73]. Similarly, we have performed VVA lectin pull-down assay as follows: 600 ug of cell lysate protein was incubated for 3 h at 4°C with 4 μg of biotinylated lectin VVA (EY Laboratories). Twenty μl of streptavidin-agarose (Sigma) was then added, and samples were incubated for an additional 2 h at 4°C with rotation. Lectin/glycoprotein complexes were collected by brief centrifugation (1400 rpm, 5 min), and washed three times with lysis buffer, followed by one wash with phosphate-buffered saline (PBS). Glycoproteins were released from the complexes by boiling in 30–50 μ1 SDS-PAGE sample buffers (5 min). The glycoproteins were resolved by SDS-PAGE, then immunoblotted to detect *MUC1* or GalNAc-conjugated proteins.

## Supplementary Figures and Table





## References

[1] Jemal A, Siegel R, Xu J, Ward E (2010). Cancer statistics, 2010. CA Cancer J Clin.

[2] Marchetti C, Pisano C, Facchini G, Bruni GS, Magazzino FP, Losito S, Pignata S (2010). First-line treatment of advanced ovarian cancer: current research and perspectives. Expert review of anticancer therapy.

[3] Jones PA, Baylin SB (2007). The epigenomics of cancer. Cell.

[4] Balch C, Fang F, Matei DE, Huang TH, Nephew KP (2009). Minireview: epigenetic changes in ovarian cancer. Endocrinology.

[5] Momparler RL (2003). Cancer epigenetics. Oncogene.

[6] Maier S, Dahlstroem C, Haefliger C, Plum A, Piepenbrock C (2005). Identifying DNA methylation biomarkers of cancer drug response. American journal of pharmacogenomics: genomics-related research in drug development and clinical practice.

[7] Szyf M, Pakneshan P, Rabbani SA (2004). DNA demethylation and cancer: therapeutic implications. Cancer letters.

[8] Bauerschlag DO, Ammerpohl O, Brautigam K, Schem C, Lin Q, Weigel MT, Hilpert F, Arnold N, Maass N, Meinhold-Heerlein I, Wagner W (2011). Progression-free survival in ovarian cancer is reflected in epigenetic DNA methylation profiles. Oncology.

[9] Watts GS, Futscher BW, Holtan N, Degeest K, Domann FE, Rose SL (2008). DNA methylation changes in ovarian cancer are cumulative with disease progression and identify tumor stage. BMC medical genomics.

[10] Li M, Balch C, Montgomery JS, Jeong M, Chung JH, Yan P, Huang TH, Kim S, Nephew KP (2009). Integrated analysis of DNA methylation and gene expression reveals specific signaling pathways associated with platinum resistance in ovarian cancer. BMC medical genomics.

[11] Keita M, Wang ZQ, Pelletier JF, Bachvarova M, Plante M, Gregoire J, Renaud MC, Mes-Masson AM, Paquet ER, Bachvarov D (2013). Global methylation profiling in serous ovarian cancer is indicative for distinct aberrant DNA methylation signatures associated with tumor aggressiveness and disease progression. Gynecologic oncology.

[12] Bennett EP, Mandel U, Clausen H, Gerken TA, Fritz TA, Tabak LA (2012). Control of mucin-type O-glycosylation: a classification of the polypeptide GalNAc-transferase gene family. Glycobiology.

[13] Hang HC, Bertozzi CR (2005). The chemistry and biology of mucin-type O-linked glycosylation. Bioorganic … medicinal chemistry.

[14] Madsen CB, Petersen C, Lavrsen K, Harndahl M, Buus S, Clausen H, Pedersen AE, Wandall HH (2012). Cancer associated aberrant protein O-glycosylation can modify antigen processing and immune response. PloS one.

[15] Wang L, Ma J, Liu F, Yu Q, Chu G, Perkins AC, Li Y (2007). Expression of MUC1 in primary and metastatic human epithelial ovarian cancer and its therapeutic significance. Gynecologic oncology.

[16] Yuan Z, Wong S, Borrelli A, Chung MA (2007). Down-regulation of MUC1 in cancer cells inhibits cell migration by promoting E-cadherin/catenin complex formation. Biochemical and biophysical research communications.

[17] Nomoto M, Izumi H, Ise T, Kato K, Takano H, Nagatani G, Shibao K, Ohta R, Imamura T, Kuwano M, Matsuo K, Yamada Y, Itoh H, Kohno K (1999). Structural basis for the regulation of UDP-N-acetyl-alpha-D-galactosamine: polypeptide N-acetylgalactosaminyl transferase-3 gene expression in adenocarcinoma cells. Cancer research.

[18] Ozaki T, Nakagawara A (2011). p53: the attractive tumor suppressor in the cancer research field. Journal of biomedicine … biotechnology.

[19] Kim DJ, Akiyama TE, Harman FS, Burns AM, Shan W, Ward JM, Kennett MJ, Gonzalez FJ, Peters JM (2004). Peroxisome proliferator-activated receptor beta (delta)-dependent regulation of ubiquitin C expression contributes to attenuation of skin carcinogenesis. The Journal of biological chemistry.

[20] Su B, Bu Y, Engelberg D, Gelman IH (2010). SSeCKS/Gravin/AKAP12 inhibits cancer cell invasiveness and Chemotaxis by suppressing a protein kinase C- Raf/MEKTERK pathway. The Journal of biological chemistry.

[21] Zhang LL, Shao SL, Wu Y (2010). [Expressions of osteopontin and B7-H4 in epithelial ovarian neoplasm and their significance]. Chinese journal of cancer.

[22] Lee KB, Byun HJ, Park SH, Park CY, Lee SH, Rho SB (2012). CYR61 controls p53 and NF-kappaB expression through PI3K/Akt/mTOR pathways in carboplatin-induced ovarian cancer cells. Cancer letters.

[23] Reuveni H, Flashner-Abramson E, Steiner L, Makedonski K, Song R, Shir A, Herlyn M, Bar-Eli M, Levitzki A (2013). Therapeutic destruction of insulin receptor substrates for cancer treatment. Cancer research.

[24] Chakrabarty S, Kondratick L (2006). Insulin-like growth factor binding protein-2 stimulates proliferation and activates multiple cascades of the mitogen-activated protein kinase pathways in NIH-OVCAR3 human epithelial ovarian cancer cells. Cancer biology … therapy.

[25] Agarwal A, Tressel SL, Kaimal R, Balla M, Lam FH, Covic L, Kuliopulos A (2010). Identification of a metalloprotease-chemokine signaling system in the ovarian cancer microenvironment: implications for antiangiogenic therapy. Cancer research.

[26] Al-Alem LF, McCord LA, Southard RC, Kilgore MW, Curry TE (2013). Activation of the PKC pathway stimulates ovarian cancer cell proliferation, migration, and expression of MMP7 and MMP10. Biology of reproduction.

[27] McLean K, Gong Y, Choi Y, Deng N, Yang K, Bai S, Cabrera L, Keller E, McCauley L, Cho KR, Buckanovich RJ (2011). Human ovarian carcinoma-associated mesenchymal stem cells regulate cancer stem cells and tumorigenesis via altered BMP production. The Journal of clinical investigation.

[28] Theriault BL, Nachtigal MW (2011). Human ovarian cancer cell morphology, motility, and proliferation are differentially influenced by autocrine TGFbeta superfamily signalling. Cancer letters.

[29] Theriault BL, Shepherd TG, Mujoomdar ML, Nachtigal MW (2007). BMP4 induces EMT and Rho GTPase activation in human ovarian cancer cells. Carcinogenesis.

[30] Do TV, Kubba LA, Du H, Sturgis CD, Woodruff TK (2008). Transforming growth factor-beta1, transforming growth factor-beta2, and transforming growth factor-beta3 enhance ovarian cancer metastatic potential by inducing a Smad3-dependent epithelial-to-mesenchymal transition. Molecular cancer research : MCR.

[31] Au CW, Siu MK, Liao X, Wong ES, Ngan HY, Tarn KF, Chan DC, Chan QK, Cheung AN (2009). Tyrosine kinase B receptor and BDNF expression in ovarian cancers - Effect on cell migration, angiogenesis and clinical outcome. Cancer letters.

[32] Zhu M, Fejzo MS, Anderson L, Dering J, Ginther C, Ramos L, Gasson JC, Karlan BY, Slamon DJ (2010). Periostin promotes ovarian cancer angiogenesis and metastasis. Gynecologic oncology.

[33] Furuya M (1999). Analysis of matrix metalloproteinases and related tissue inhibitors in cystic fluids of ovarian tumors. [Hokkaido igaku zasshi]. The Hokkaido journal of medical science.

[34] Hernandez L, Hsu SC, Davidson B, Birrer MJ, Kohn EC, Annunziata CM (2010). Activation of NF-KB Signaling by Inhibitor of NF-κB Kinase β Increases Aggressiveness of Ovarian Cancer. Cancer research.

[35] Kitada S, Yamada S, Kuma A, Ouchi S, Tasaki T, Nabeshima A, Noguchi H, Wang KY, Shimajiri S, Nakano R, Izumi H, Kohno K, Matsumoto T, Sasaguri Y (2013). Polypeptide N-acetylgalactosaminyl transferase 3 independently predicts high-grade tumours and poor prognosis in patients with renal cell carcinomas. British journal of cancer.

[36] Mochizuki Y, Ito KI, Izumi H, Kohno K, Amano J (2013). Expression of Polypeptide N-acetylgalactosaminyl Transferase-3 and Its Association with Clinicopathological Factors in Thyroid Carcinomas. Thyroid : official journal of the American Thyroid Association.

[37] Taniuchi K, Cerny RL, Tanouchi A, Kohno K, Kotani N, Honke K, Saibara T, Hollingsworth MA (2011). Overexpression of GalNAc-transferase GalNAc-T3 promotes pancreatic cancer cell growth. Oncogene.

[38] Inoue T, Eguchi T, Oda Y, Nishiyama K, Fujii K, Izumi H, Kohno K, Yamaguchi K, Tanaka M, Tsuneyoshi M (2007). Expression of GalNAc-T3 and its relationships with clinicopathological factors in 61 extrahepatic bile duct carcinomas analyzed using stepwise sections - special reference to its association with lymph node metastases. Modern pathology : an official journal of the United States and Canadian Academy of Pathology, Inc.

[39] Onitsuka K, Shibao K, Nakayama Y, Minagawa N, Hirata K, Izumi H, Matsuo K, Nagata N, Kitazato K, Kohno K, Itoh H (2003). Prognostic significance of UDP-N-acetyl-alpha-D-galactosamine:polypeptide N-acetylgalactosaminyltransferase-3 (GalNAc-T3) expression in patients with gastric carcinoma. Cancer science.

[40] Dosaka-Akita H, Kinoshita I, Yamazaki K, Izumi H, Itoh T, Katoh H, Nishimura M, Matsuo K, Yamada Y, Kohno K (2002). N-acetylgalactosaminyl transferase-3 is a potential new marker for non-small cell lung cancers. British journal of cancer.

[41] Shibao K, Izumi H, Nakayama Y, Ohta R, Nagata N, Nomoto M, Matsuo K, Yamada Y, Kitazato K, Itoh H, Kohno K (2002). Expression of UDP-N-acetyl-alpha-D-galactosamine-polypeptide galNAc N-acetylgalactosaminyl transferase-3 in relation to differentiation and prognosis in patients with colorectal carcinoma. Cancer.

[42] Kato K, Takeuchi H, Kanoh A, Miyahara N, Nemoto-Sasaki Y, Morimoto-Tomita M, Matsubara A, Ohashi Y, Waki M, Usami K, Mandel U, Clausen H, Higashi N, Irimura T (2010). Loss of UDP-GalNAc:polypeptide N-acetylgalactosaminyltransferase 3 and reduced O-glycosylation in colon carcinoma cells selected for hepatic metastasis. Glycoconjugate journal.

[43] Topaz O, Shurman DL, Bergman R, Indelman M, Ratajczak P, Mizrachi M, Khamaysi Z, Behar D, Petronius D, Friedman V, Zelikovic I, Raimer S, Metzker A, Richard G, Sprecher E (2004). Mutations in GALNT3, encoding a protein involved in O-linked glycosylation, cause familial tumoral calcinosis. Nature genetics.

[44] Park JH, Nishidate T, Kijima K, Ohashi T, Takegawa K, Fujikane T, Hirata K, Nakamura Y, Katagiri T (2010). Critical roles of mucin 1 glycosylation by transactivated polypeptide N-acetylgalactosaminyltransferase 6 in mammary carcinogenesis. Cancer research.

[45] Gomes J, Marcos NT, Berois N, Osinaga E, Magalhaes A, Pinto-de-Sousa J, Almeida R, Gartner F, Reis CA (2009). Expression of UDP-N-acetyl-D-galactosamine: polypeptide N-acetylgalactosaminyltransferase-6 in gastric mucosa, intestinal metaplasia, and gastric carcinoma. The journal of histochemistry and cytochemistry : official journal of the Histochemistry Society.

[46] Matsumoto Y, Zhang Q, Akita K, Nakada H, Hamamura K, Tokuda N, Tsuchida A, Matsubara T, Hori T, Okajima T, Furukawa K, Urano T (2012). pp-GalNAc-T13 induces high metastatic potential of murine Lewis lung cancer by generating trimeric Tn antigen. Biochemical and biophysical research communications.

[47] Berois N, Blanc E, Ripoche H, Mergui X, Trajtenberg F, Cantais S, Barrois M, Dessen P, Kagedal B, Benard J, Osinaga E, Raguenez G (2006). ppGalNAc-T13: a new molecular marker of bone marrow involvement in neuroblastoma. Clinical chemistry.

[48] Wang R, Yu C, Zhao D, Wu M, Yang Z (2013). The mucin-type glycosylating enzyme polypeptide N-acetylgalactosaminyltransferase 14 promotes the migration of ovarian cancer by modifying mucin 13. Oncology reports.

[49] Ding MX, Wang HF, Wang JS, Zhan H, Zuo YG, Yang DL, Liu JY, Wang W, Ke CX, Yan RP (2012). ppGalNAc T1 as a potential novel marker for human bladder cancer. Asian Pacific journal of cancer prevention : APJCP.

[50] Gao Y, Liu Z, Feng J, Sun Q, Zhang B, Zheng W, Ma W (2013). Expression pattern of polypeptide N-acetylgalactosaminyltransferase-10 in gastric carcinoma. Oncology letters.

[51] Hua D, Shen L, Xu L, Jiang Z, Zhou Y, Yue A, Zou S, Cheng Z, Wu S (2012). Polypeptide N-acetylgalactosaminyltransferase 2 regulates cellular metastasis-associated behavior in gastric cancer. International journal of molecular medicine.

[52] Liu J, Yang L, Jin M, Xu L, Wu S (2011). regulation of the invasion and metastasis of human glioma cells by polypeptide N-acetylgalactosaminyltransferase 2. Molecular medicine reports.

[53] Wu YM, Liu CH, Hu RH, Huang MJ, Lee JJ, Chen CH, Huang J, Lai HS, Lee PH, Hsu WM, Huang HC, Huang MC (2011). Mucin glycosylating enzyme GALNT2 regulates the malignant character of hepatocellular carcinoma by modifying the EGF receptor. Cancer research.

[54] Berois N, Gattolliat CH, Barrios E, Capandeguy L, Douc-Rasy S, Valteau-Couanet D, Benard J, Osinaga E (2013). GALNT9 gene expression is a prognostic marker in neuroblastoma patients. Clinical chemistry.

[55] Guo JM, Chen HL, Wang GM, Zhang YK, Narimatsu H (2004). Expression of UDP-GalNAc:polypeptide N-acetylgalactosaminyltransferase-12 in gastric and colonic cancer cell lines and in human colorectal cancer. Oncology.

[56] Wagner KW, Punnoose EA, Januario T, Lawrence DA, Pitti RM, Lancaster K, Lee D, von Goetz M, Yee SF, Totpal K, Huw L, Katta V, Cavet G, Hymowitz SG, Amler L, Ashkenazi A (2007). Death-receptor O-glycosylation controls tumor-cell sensitivity to the proapoptotic ligand Apo2L/TRAIL. Nature medicine.

[57] Peng RQ, Wan HY, Li HF, Liu M, Li X, Tang H (2012). MicroRNA-214 suppresses growth and invasiveness of cervical cancer cells by targeting UDP-N-acetyl-alpha-D-galactosamine:polypeptide N-acetylgalactosaminyltransferase 7. The Journal of biological chemistry.

[58] Gaziel-Sovran A, Segura MF, Di Micco R, Collins MK, Hanniford D, Vega-Saenz de Miera E, Rakus JF, Dankert JF, Shang S, Kerbel RS, Bhardwaj N, Shao Y, Darvishian F, Zavadil J, Erlebacher A, Mahal LK (2011). miR-30b/30d regulation of GalNAc transferases enhances invasion and immunosuppression during metastasis. Cancer cell.

[59] Li Z, Yamada S, Inenaga S, Imamura T, Wu Y, Wang KY, Shimajiri S, Nakano R, Izumi H, Kohno K, Sasaguri Y (2011). Polypeptide N-acetylgalactosaminyltransferase 6 expression in pancreatic cancer is an independent prognostic factor indicating better overall survival. British journal of cancer.

[60] Meany DL, Chan DW (2011). Aberrant glycosylation associated with enzymes as cancer biomarkers. Clinical proteomics.

[61] Ten Hagen KG, Fritz TA, Tabak LA (2003). All in the family: the UDP-GalNAc: polypeptide N-acetylgalactosaminyltransferases. Glycobiology.

[62] Carraway KL, Funes M, Workman HC, Sweeney C (2007). Contribution of membrane mucins to tumor progression through modulation of cellular growth signaling pathways. Current topics in developmental biology.

[63] Burford B, Gentry-Maharaj A, Graham R, Allen D, Pedersen JW, Nudelman AS, Blixt O, Fourkala EO, Bueti D, Dawnay A, Ford J, Desai R, David L, Trinder P, Acres B, Schwientek T (2013). Autoantibodies to MUC1 glycopeptides cannot be used as a screening assay for early detection of breast, ovarian, lung or pancreatic cancer. British journal of cancer.

[64] Deng J, Wang L, Chen H, Li L, Ma Y, Ni J, Li Y (2013). The role of tumour-associated MUC1 in epithelial ovarian cancer metastasis and progression. Cancer metastasis reviews.

[65] Inoue M, Takahashi S, Yamashina I, Kaibori M, Okumura T, Kamiyama Y, Vichier-Guerre S, Cantacuzene D, Nakada H (2001). High density O-glycosylation of the MUC2 tandem repeat unit by N-acetylgalactosaminyltransferase-3 in colonic adenocarcinoma extracts. Cancer research.

[66] Wandall HH, Hassan H, Mirgorodskaya E, Kristensen AK, Roepstorff P, Bennett EP, Nielsen PA, Hollingsworth MA, Burchell J, Taylor-Papadimitriou J, Clausen H (1997). Substrate specificities of three members of the human UDP-N-acetyl-alpha-D-galactosamine:Polypeptide N-acetylgalactosaminyltransferase family, GalNAc-T1, -T2, and -T3. The Journal of biological chemistry.

[67] Bennett EP, Hassan H, Mandel U, Hollingsworth MA, Akisawa N, Ikematsu Y, Merkx G, van Kessel AG, Olofsson S, Clausen H (1999). Cloning and characterization of a close homologue of human UDP-N-acetyl-alpha-D-galactosamine:Polypeptide N-acetylgalactosaminyltransferase-T3, designated GalNAc-T6. Evidence for genetic but not functional redundancy. The Journal of biological chemistry.

[68] Taylor PT, Haverstick D (2005). Re: New guidelines to evaluate the response to treatment in solid tumors (ovarian cancer). Journal of the National Cancer Institute.

[69] Keita M, Bachvarova M, Morin C, Plante M, Gregoire J, Renaud MC, Sebastianelli A, Trinh XB, Bachvarov D (2013). The RUNX1 transcription factor is expressed in serous epithelial ovarian carcinoma and contributes to cell proliferation, migration and invasion. Cell cycle.

[70] Mercier PL, Bachvarova M, Plante M, Gregoire J, Renaud MC, Ghani K, Tetu B, Bairati I, Bachvarov D (2011). Characterization of DOK1, a candidate tumor suppressor gene, in epithelial ovarian cancer. Molecular oncology.

[71] Wang ZQ, Keita M, Bachvarova M, Gobeil S, Morin C, Plante M, Gregoire J, Renaud MC, Sebastianelli A, Trinh XB, Bachvarov D (2013). Inhibition of RUNX2 Transcriptional Activity Blocks the Proliferation, Migration and Invasion of Epithelial Ovarian Carcinoma Cells. PloS one.

[72] Tollefsen SE, Kornfeld R (1983). Isolation and characterization of lectins from Vicia villosa. Two distinct carbohydrate binding activities are present in seed extracts. The Journal of biological chemistry.

[73] Qiu Y, Patwa TH, Xu L, Shedden K, Misek DE, Tuck M, Jin G, Ruffin MT, Turgeon DK, Synal S, Bresalier R, Marcon N, Brenner DE, Lubman DM (2008). Plasma glycoprotein profiling for colorectal cancer biomarker identification by lectin glycoarray and lectin blot. Journal of proteome research.

