# Supplementary material for: Role of the polypeptide N-acetylgalactosaminyltransferase 3 in ovarian cancer progression: possible implications in abnormal mucin O-glycosylation
Source: Oncotarget. 2014 Jan 27;5(2):544–60. doi: 10.18632/oncotarget.1652 (PMC3964228; doi:10.18632/oncotarget.1652)
Supplement: Supplementary file 1 [file oncotarget-05-544-s001.pdf]

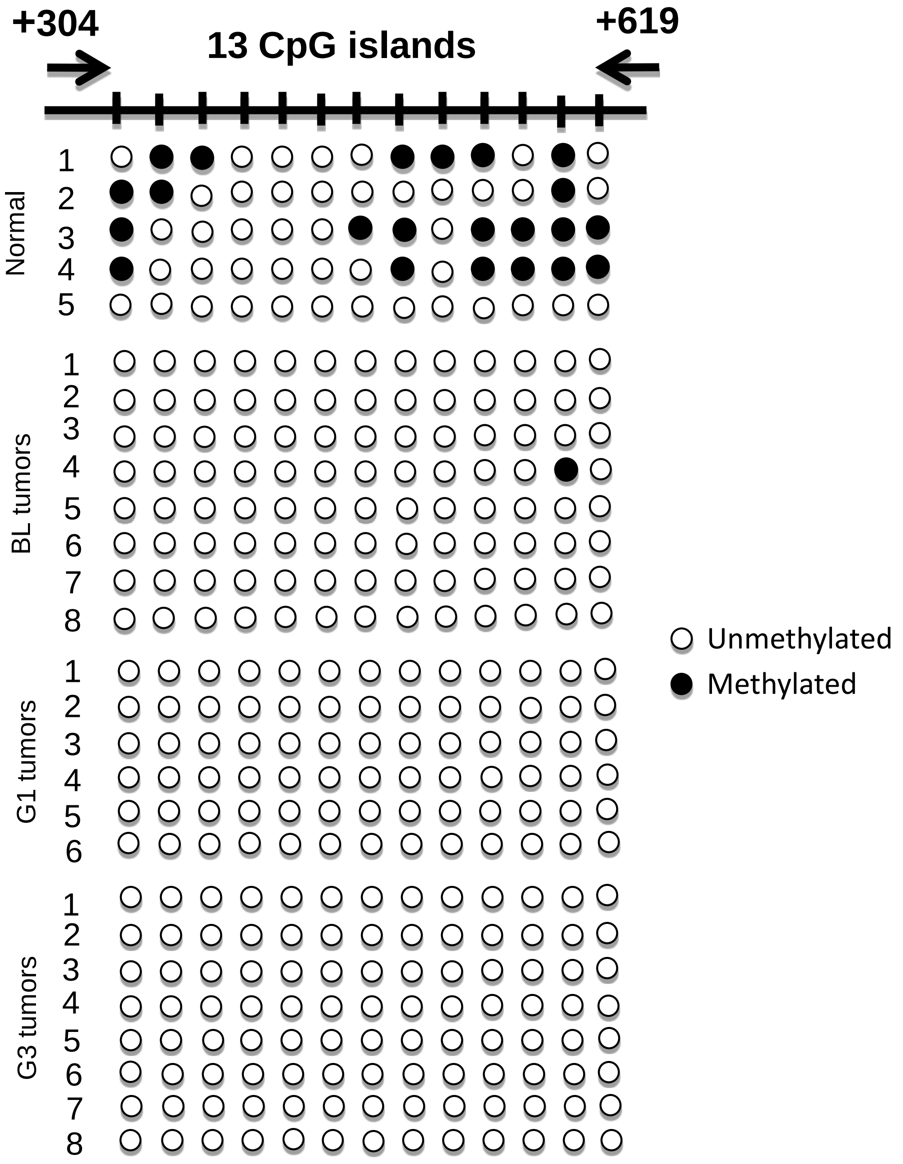

Supplemental Figure 1. BSP analysis of the methylation status of GALNT3 in borderline (BL), grade 1 (G1) and grade 3 (G3) EOC tumors compared to normal ovarian tissues. Filled circles represent methylated CpGs and open circles represent unmethylated CpGs. CpG plot of the analyzed region is also presented (CpGs are displayed with vertical marks). The indicated positions on the CpG plot represent the number of nucleotides stretching downstream of the first exon of the GALNT3 gene.

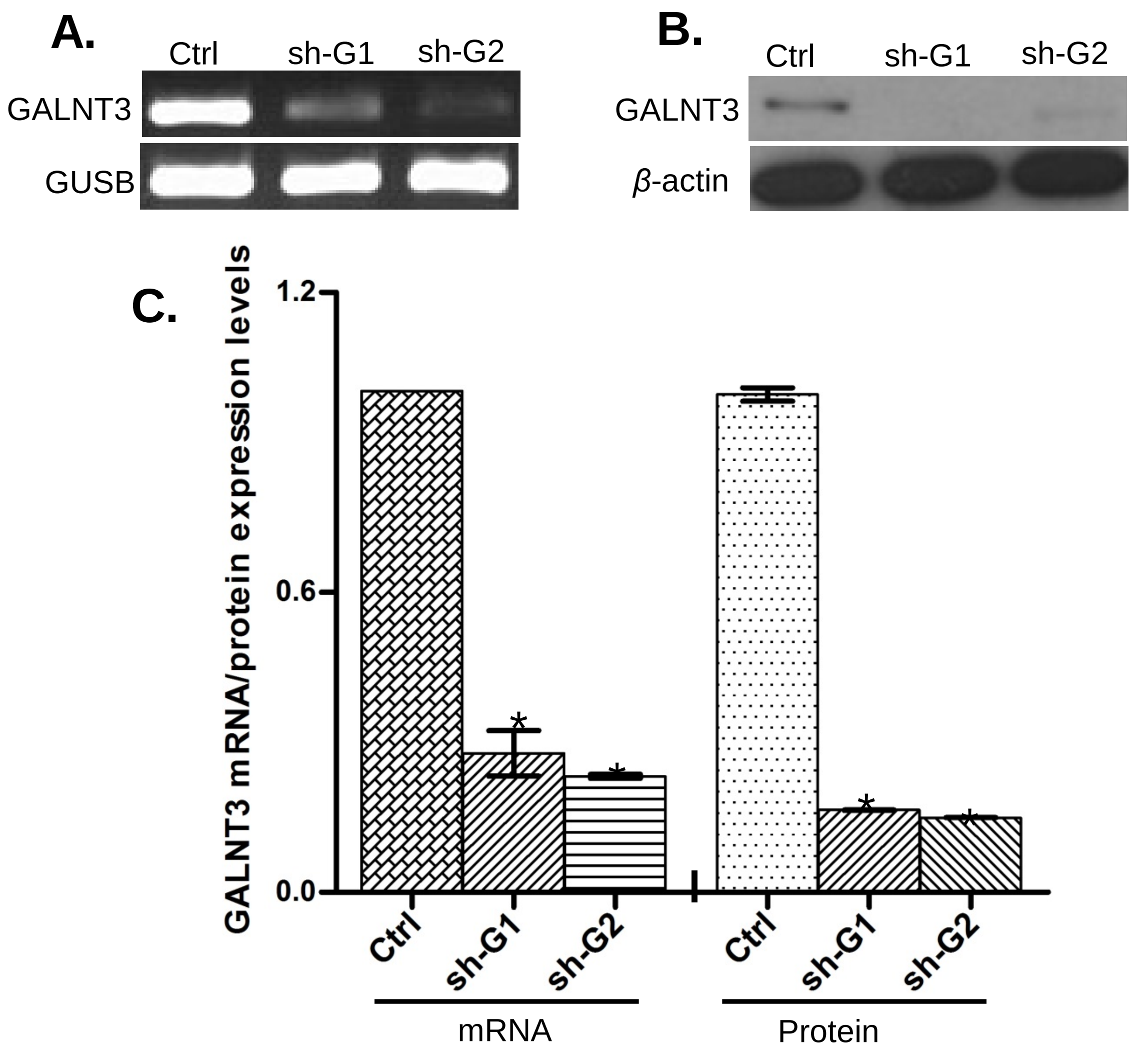

Supplemental Figure 2. Analysis of GALNT3 expression in A2780s cells. A. Semi-quantitative duplex RT-PCR (sqRT-PCR) analysis of GALNT3 mRNA expression levels in the shRNA-GALNT3 clones 1 and 2 (sh-G1 and sh-G2), compared to the mock-transfected control clone (ctrl). Displayed are images of representative results following sqRT-PCR analysis. The GUSB gene was used as internal standard. B. Western-blot analysis of GALNT3 protein expression in the shRNA-GALNT3 clones 1 and 2 (sh-G1 and sh-G2), compared to the control clone (ctrl). β-actin was used as a loading control.

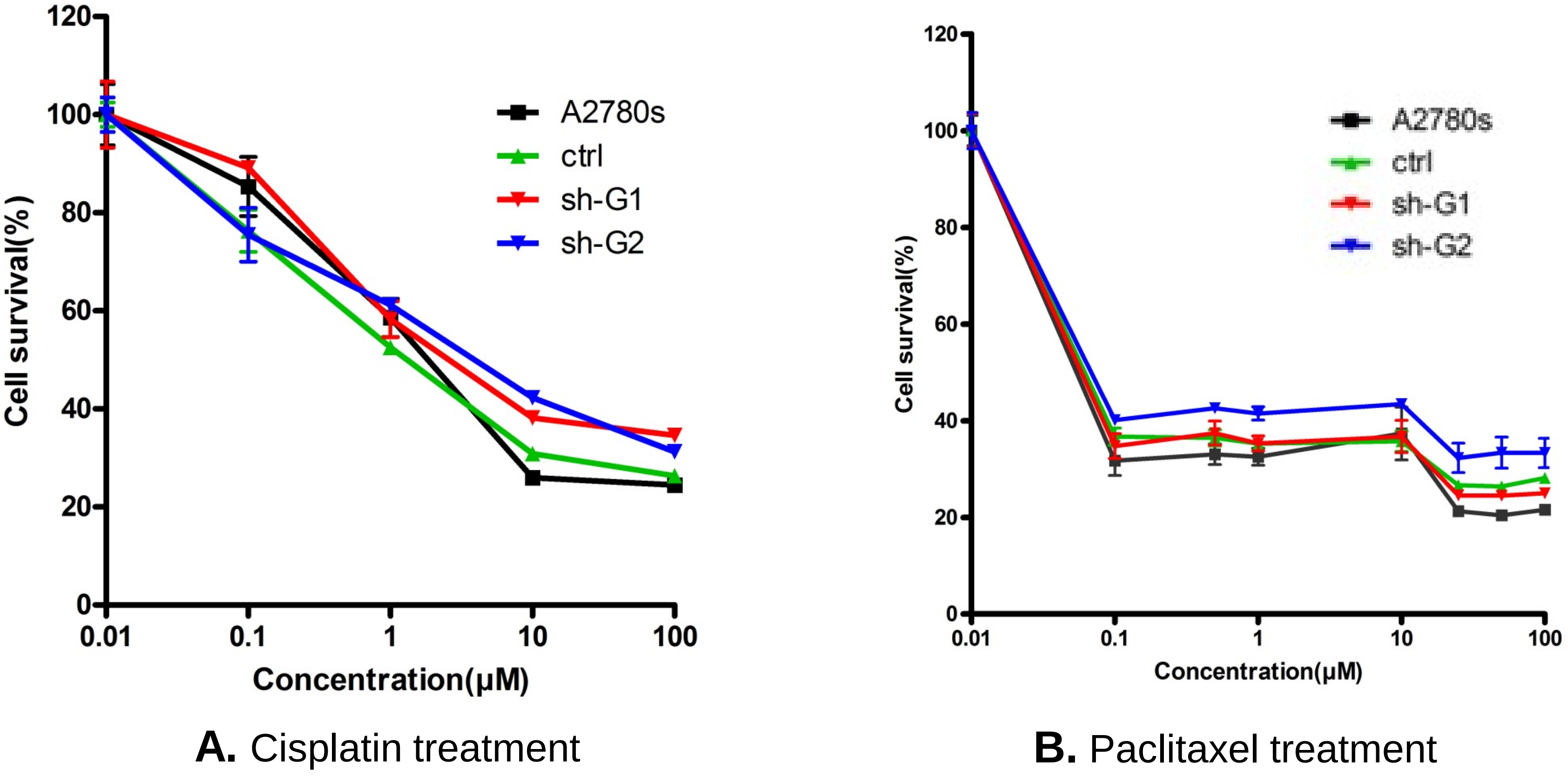

Supplemental Figure 3. Dose-response cytotoxicity curves upon cisplatin (A) and paclitaxel (B) treatment of A2780s cells following shRNA-mediated GALNT3 knockdown. Treatment responses of the shRNA-GALNT3 clones sh-G1 and sh-G2 were compared to to the mock-transfected control (ctrl) clone. Error bars denote ± SEM. All the *P* value are > 0.05 according to the one-way ANOVA analysis.
